# Determination of paramagnetic ferrous gel sensitivity in low energy x-ray beam produced by a miniature accelerator

**DOI:** 10.1371/journal.pone.0232315

**Published:** 2020-05-04

**Authors:** Yassir Ben Ahmed, Jérémy Coulaud, Soléakhena Ken, Laure Parent

**Affiliations:** 1 Département d’Ingénierie et de Physique médicale, Institut Claudius Regaud (ICR), Institut Universitaire du Cancer de Toulouse-Oncopole (IUCT-O), Toulouse, France; 2 SIMAD LU50, Université Paul Sabatier Toulouse III, Castres, Toulouse, France; Stanford University School of Medicine, UNITED STATES

## Abstract

The INTRABEAM Carl Zeiss Surgical system (Oberkochen, Germany) is a miniature accelerator producing low energy photons (50 keV maximum). The published dosimetric characterization of the INTRABEAM was based on detectors (radiochromic films or ionization chambers) not allowing measuring the absorbed dose in the first millimeters of the irradiated medium, where the dose is actually prescribed. This study aims at determining with Magnetic Resonance Imaging (MRI) the sensitivity of a paramagnetic gel in order to measure the dose deposit produced with the INTRABEAM from 0 to 20 mm. Although spherical applicators are mostly used with the INTRABEAM system for breast applications, this study focuses on surface applicators that are of interest for cutaneous carcinomas. The irradiations at 12 different dose levels (between 2 Gy and 50 Gy at the gel surface) were performed with the INTRABEAM and a 4 cm surface applicator. The gel used in this study is a new « sensitive » material. In order to compare gel sensitivity at low energy with high energy, gels were irradiated by an 18 MV photon beam produced by a Varian Clinac 2100 CD. T_2_ weighted multi echo MRI sequences were performed with 16 echo times. The T_2_ signal versus echo times was fitted with a mono-exponential function with 95% confidence interval. The calibration curve determined at low energy is a linear function (r^2^ = 0.9893) with a sensitivity of 0.0381 s^-1^.Gy^-1^, a similar linear function was obtained at high energy (0.0372 s^-1^.Gy^-1^ with r^2^ = 0.9662). The calibration curve at low energy was used to draw isodose maps from the MR images. The PDD (Percent Depth Dose) determined in the gel is within 5%-1mm of the ionization chamber PDD except for one point. The dosimetric sensitivity of this new paramagnetic ferrous gel was determined with MRI measurements. It allowed measuring the dose distribution specifically in the first millimeters for an irradiation with the INTRABEAM miniature accelerator equipped with a surface applicator.

## Introduction

Among detectors available to measure dose distribution, very few are capable of 3D dose measurements. Paramagnetic gels have proven their relevance in the determination of the 3D dose distribution and their use has spread since several years [1–5]. Dose distribution deposited by an irradiation in such gels can be measured by optical Computed Tomography (OCT), where the quantification is based on intensity difference of laser beams through the gels [6–8] or by magnetic resonance (MR) imaging [9,10]. Gels are used as chemical dosimeters and phantom at the same time and, in our study, the dose is determined from a measurable change on the MR signal impacted by the chemical state of the gel. The characterization of these chemical reactions enables to define gels as dosimeters. Gels record and preserve the spatial distribution information of the absorbed dose. One of the benefits of the gel relative to other dosimeters is its tissue equivalent density and the possibility to shape the gel as an anthropomorphic phantom for example.

Studies on gel, measured by MR after irradiation, described mainly two kinds of paramagnetic gels: polymer gels [10,11] and ferrous gels (also named Fricke gels) [12,13]. In polymer gels, the irradiation induces the polymerization of the monomers. The polymer concentration increases and the relaxivity, related to the mobility of the water molecules, increases proportionally to the absorbed dose. Polymer gels are thermodynamically stable but they are extremely sensitive to light and oxygen [14]. If not carefully used, they might be prone to loss in resolution, because the polymerization does not stop immediately following irradiation and continued polymerization results in loss of spatial resolution. In ferrous gels, under the effect of irradiation, Fe2+ (ferrous ion) is oxidized to Fe3+ (ferric ion) after radiolysis of the water in the presence of oxygen [15]. The concentration of ferric ions modifies the gel relaxivity, proportionally to the absorbed dose [2]. Less expensive and non-toxic, ferrous gels are easier to manipulate than polymer gels [16]. Nevertheless, the ferrous gels are not widely used. The main drawback of these dosimeters is the diffusion of the paramagnetic ions inside the gels. The diffusion depends on the storage duration and temperature [17] and results in a loss of spatial information [18].

Recently a preparation of a stabilized dosimetric gelatin (EasyDosit, MCP, France) was used to prepare radiosensitive phantoms with different equivalent tissue properties and to carry out preliminary studies in radiodosimetry [19–21]. After irradiation of gel dosimeters, change of the gel magnetic properties can be detected by measuring the spin-lattice (T_1_) and spin-spin (T_2_) relaxation constants from the MR images [16]. It was demonstrated that T_2_ is a better candidate to detect radiochemical reactions due to its higher sensitivity to polymerization. Indeed, T_2_ relaxation is an indirect measure of the absorbed dose due to the ionization radiation in those gels [22]. Relaxivity is defined as the inverse of relaxation time (R = 1/T). Both R_1_ and R_2_ relaxivity are proportional to the absorbed dose [17], and the dose distribution can be determined with 3D MR images of the irradiated gel. Nonetheless the linear model was found to be not reliable for low and high doses [23]. The most common method for gel calibration consists of irradiating the gel at different known dose levels and measuring the R_2_ average value in the corresponding region of interest (ROI) [12,14].

The Carl Zeiss surgical INTRABEAM^®^ system (Oberkochen, Germany) is a miniature accelerator producing low energy photons (50 keV maximum). It is mostly used for breast targeted intraoperative therapy (TARGIT) with spherical applicators [24,25] but surface and flat applicators have been developed for other applications [26]. Without applicators, the beam half value layer (HVL) is 0.43 mm Al [27]. Surface applicators are preferably used for cutaneous carcinomas while flat applicators are used for intraoperative treatments [26]. Both applicators convert the spherical absorbed dose distribution in a flat absorbed dose distribution [27]. The mean energy of the beam for flat and surface applicators is in the order of 30 keV [28]. The dose rate at the surface can be up to 30 Gy/min but decreases rapidly with depth (almost 0 Gy/min at 20 mm depth). Depending on the applicator diameter, the dose is divided by two after 1 to 2 mm for surface applicators and after 1 to 6 mm for flat applicators. Moreover, the smaller the applicator diameter, the higher the dose rate [27].

This study focuses on surface applicators as they are easier to handle with gels and also because the dose gradient with depth is the steepest among available applicators, making dose measurements more challenging.

The literature on dosimetric characterization of the INTRABEAM surface applicators is mostly based on radiochromic measurements or ionization chambers [26,27,29]. Ionization chamber is the reference detector for absolute dosimetry. At this energy range, international dosimetry protocols recommend the use of plane-parallel chambers [30,31]. The effective point of measurement is not at the surface of the chamber. Furthermore, for water measurements, a waterproof sleeve is necessary around the ionization chamber as it is not waterproof. It is therefore not possible to measure the absorbed dose in the first two millimeters. With radiochromic films, it is difficult to accurately measure the dose in the direction of the applicator axis: an overestimation of the dose might occur because of the incident directional dependence of the films [29,32] and possible air gaps between the film and the phantom [32,33]. Furthermore, measurements in the first millimeters are subject to uncertainties as a layer of separation can appear at the edge of the film [34]. Films must therefore be placed perpendicular to the beam axis [35].

This study investigates the use of a different type of detector, the paramagnetic gels, for low energy x-ray beam dosimetry.

Most papers on ferrous gel dosimeters with MR reading are related to high energy photon external radiotherapy [15,36,37] and brachytherapy dose measurements [38,39] where photon energy range is from about 300 keV (^192^Ir sources) to 25 MeV (clinical linear accelerators).

Solc et al published a study on the INTRABEAM system and ferrous gel dosimeters with optical reading [40] but so far, no studies have been published on ferrous gel dosimeters with MR reading of X-ray sources below 50 keV.

This work aims at determining the sensitivity of a paramagnetic ferrous gel with MR reading in low energy x-ray beam using an INTRABEAM system miniature accelerator, with a maximum photon energy of 50 keV. Because the main issue in gel dosimetry at low energy is the dose gradient, in this study, a specific procedure was followed for the calibration of irradiated gel at low energies. In addition, the MR imaging sequence was optimized to obtain a sub-millimeter spatial resolution with a high signal-to-noise ratio. Gel sensitivity at low and high energy (18 MV photon beams) were also compared. The highest available energy (18 MV) was used to minimize dose gradient within the sample.

## Materials and methods

### Dosimetric gel

The gel used in this work is a new sensitive material formulated from patented products (EasyDosit, and Solifer manufactured by MCP, Toulouse, France) following the protocol outlined by Coulaud et al [19].

The gel tissue equivalence is estimated at low energy with the same methodology as in the work of J. Coulaud *et al*, including the determination of the mass attenuation coefficient, the mass energy absorption coefficient, electron mass stopping and angular scattering power ratios [19]. This gel was shown to be tissue equivalent at high energy [19].

These new gels allow controlling diffusion in order to prevent loss of resolution with time [20,41].

EasyDosit is mainly composed by porcine gelatin (270 Bloom), sucrose (20%) and some preservative (0.1%) such as mannan, beta- D-Glucan, genipin, glutaraldehyde or methylparaben, in order to avoid degradation of the gelatin. EasyDosit is stable for 1 year when stored at 4°C in plastic bags in order to avoid dehydration. EasyDosit can be liquefied at 40°C, then the required amount of water, sulfuric acid and Mohr salt can be added before pouring the mixture into the appropriate mold for dosimetry.

Gel was prepared as described by Coulaud *et al* [19,42] for the breast equivalent gel. Samples have been brought to the room temperature, 4 h before the irradiation.

### INTRABEAM low energy irradiation

The Carl Zeiss Surgical INTRABEAM system (Oberkochen, Germany) was used to carry out the irradiations. This is the same system used by Goubert et al Al [27]. HVL was measured at 0.61 mm Al with a bare probe (without applicator) and 0.43 mm Al with the 4 cm surface applicator [27], the only applicator investigated in this study.

The ferrous gel was placed in plastic containers of 6.5 cm height with a diameter of 5 cm in order to perform irradiations with the INTRABEAM system.

**Fig 1:**
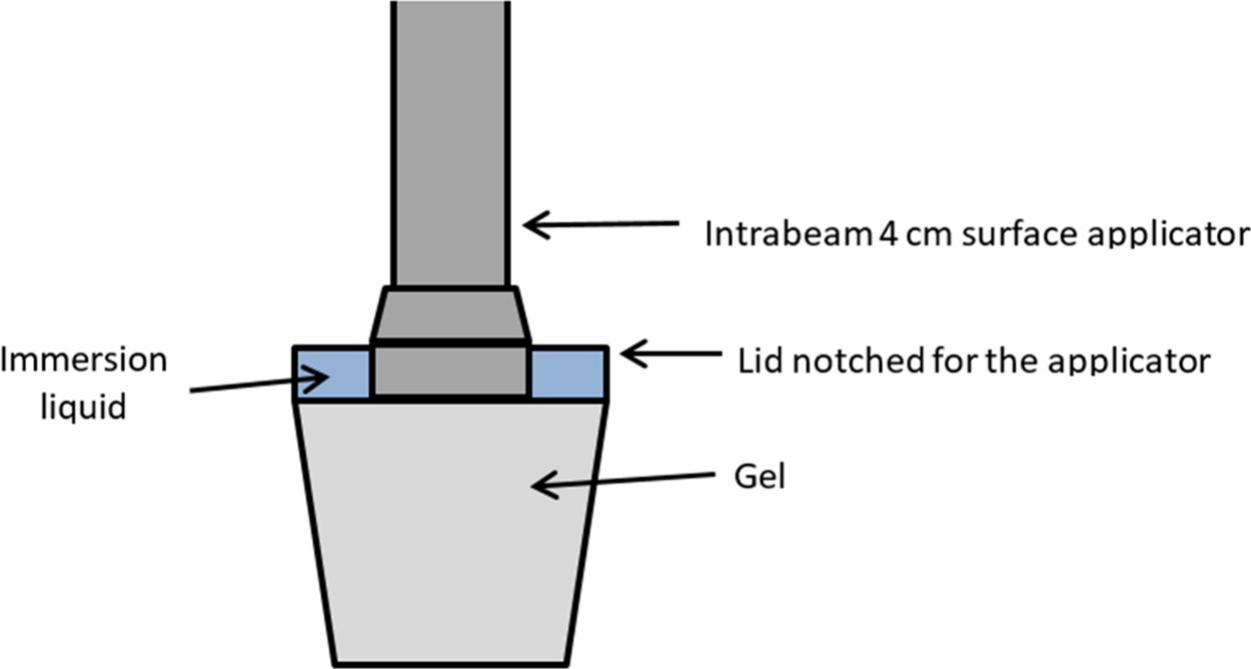
Irradiation configuration with plastic containers.

Irradiations were performed for twelve dose levels: 2 Gy, 4 Gy, 6 Gy, 8 Gy, 10 Gy, 15 Gy, 20 Gy, 25 Gy, 30 Gy, 35 Gy, 40 Gy and 50 Gy. A non-irradiated gel region at the bottom of a plastic container was used as reference. During irradiations, particular care was given to ensure that the applicator was in contact with the gel. A special irradiation configuration was set-up for that purpose (Fig 1). Without immersion liquid at the interface, the gel adhered to the metal surface of the applicator, particularly for the higher dose levels (>35Gy) for which the treatment time was important. As a result, the gel surface was ripped off when removing the applicator. The use of water as immersion liquid enabled to overcome the loss of gel surface matter for high doses (Fig 1).

### High energy irradiation

7 cm height and 3.5 cm diameter cylindrical plastic containers were used for irradiations at high energy. Irradiations were performed on a Varian Clinac 2100 CD with an 18 MV photon external beam. Six dose levels were carried out: 2.5 Gy, 5 Gy, 10 Gy, 15 Gy, 20 Gy and 25 Gy. One non-irradiated sample was used as a reference.

A homogeneous dose in the gel was obtained by placing the plastic containers in the middle of a 30x30x30 cm^3^ water tank and an irradiation configuration with parallel opposed beams of 15x15 cm^2^ at isocentre.

### MR imaging measurement

In this study, T_2_ weighted MR images were acquired for the reading of the irradiated gel. The MR imaging sequence optimization is an important step as the signal-to-noise ratio (SNR) and spatial resolution determine the reading quality of the gel. SNR was measured for MR images using the same region-of-interest on the reference, i.e. the non-irradiated batch (0 Gy). Mean SNR was computed according to the formula cited in [43] and was equal to 107.14 ± 54.09.

MR acquisitions were performed on a 1.5 T Siemens Magnetom Aera MR scan (Siemens Healthcare, Erlangen, Germany) with surface flex head coils technology. MR gel measurements were done within 3 hours after irradiation. T_2_ weighted multi echo sequences were performed to acquire 5 slices of 2 mm thickness in the left-right direction, using a 200x200 mm^2^ field of view, with 256x256 sample matrix resulting in pixel size 0.78x0.78 mm^2^ in the anterior-posterior and superior-inferior directions, adequate for sub-millimetric exploration in the sagittal orientation. Sixteen echo times (from 22.5 ms to 360 ms, separated by 22.5 ms), were acquired with TR = 2000 ms. Total acquisition time was 7 minutes.

**Fig 2:**
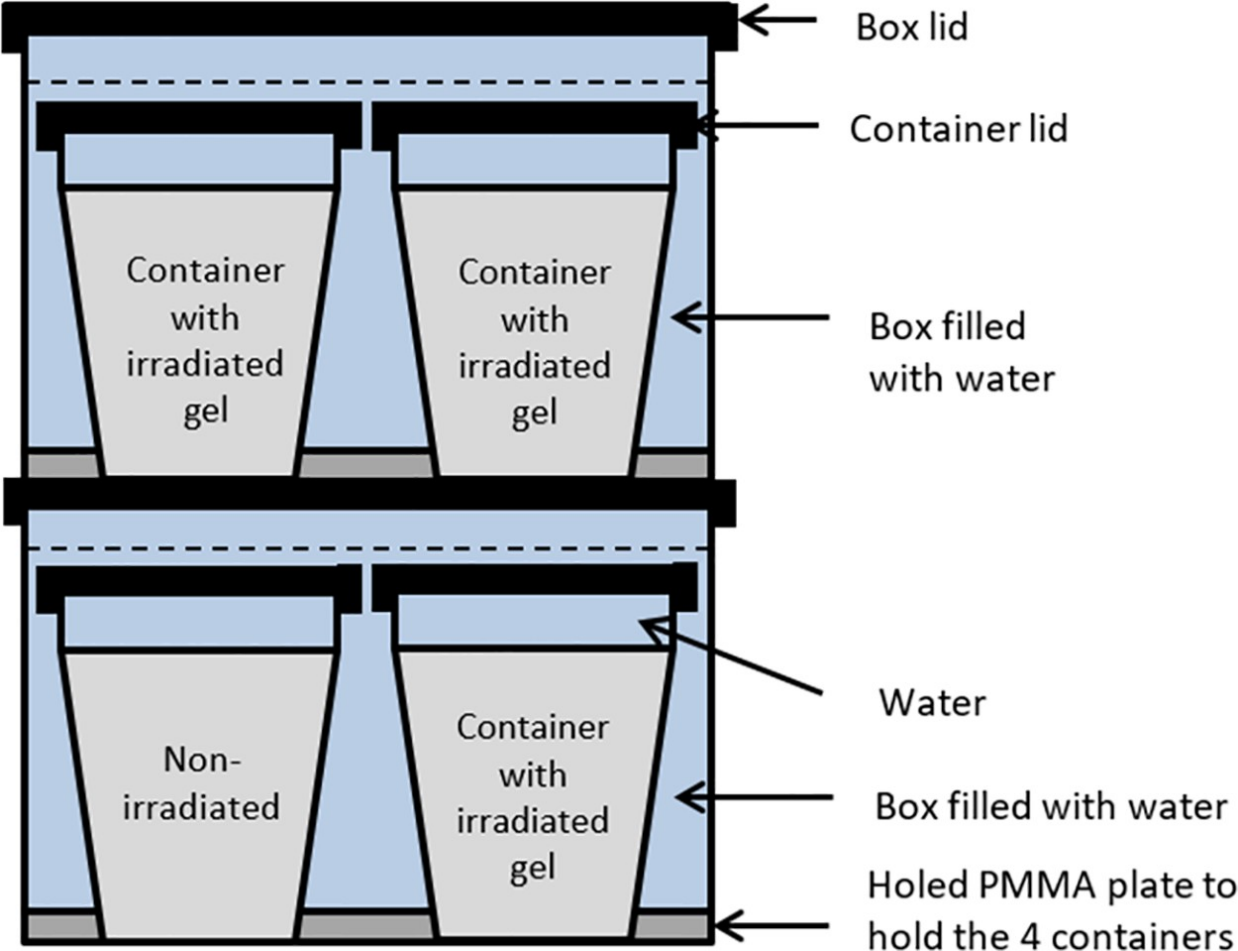
Sagittal view of the plastic containers acquisition set-up inside the MR scan.

The configuration was defined in order to perform reproducibility testing (Fig 2). For low energy irradiations, 8 plastic containers were placed in 2 superimposed plastic boxes (23x13.6x16 cm^3^), resulting in 2 levels of 4 plastic containers maintained by a holed PMMA plate. Boxes were filled with water in order to get enough proton density to apply the RF pulse and to minimize the interface artifact which is more pronounced for gel/air interface. The first sagittal MR image acquired one set of four plastic containers (two on the bottom and two on the top) including one batch with no irradiation used as reference. The sagittal acquisition was repeated for the second contralateral set of four plastic containers.

For high energy irradiation, all plastic containers were placed in a polystyrene holder. The signal, read from the middle of the sample, was not affected by surface artifacts.

For high and low energy irradiations, each plastic container was randomly moved 5 times to measure the reproducibility. The means and standard deviations of two successive scans were considered to compute the repeatability measurement in a same plastic container and dose.

The total time between the gel preparation and the last MR measurement did not exceed 6 hours which sufficiently respected the solidification time of the ferrous gel (for indication, 200 mL of ferrous gel will take 180 min to reach 20° C when cooled at room temperature but only 50 min when placed at 4° C).

### Analysis

#### Isodose map

MR images analysis were performed using Matlab (Version 7.11, MathWorks, Natrick, MA, US). Median filter was used to enhance the signal to noise ratio and reduce the ring artifact. T_2_ maps were obtained by fitting, pixel by pixel, the 16 echoes to a monoexponential function. The first echo time was not considered for the fit. The T_2_ map was converted to a dose map using a linear fit calibration (T_2_ vs dose) obtained from the plastic container calibration.

$R_{2}{}_{{}_{0}}$ average (which corresponds to the R_2_ parameter of the gel reference) and standard deviation (SD) were calculated in a non-irradiated gel region (bottom of plastic containers). An area was automatically detected at the surface to compute the average of the maximum of ${\text{R}}_{2}-R_{2}{}_{{}_{0}}$.

#### PDD (percent depth dose)

The dose response, determined from MR measurements, was established from the surface to a depth of 22 mm for an irradiation level of 50 Gy. The same INTRABEAM was already characterized and the PDD was established from measurements with a PTW 34013 ion chamber [27]. This is a plane-parallel chamber with a small sensitive volume (0.0053 cm^3^) that makes it adequate for measurements in steep gradient fluence. Note that with this chamber, the absorbed dose cannot be measured in the first two millimeters. Normalization was performed to compare the two PDD. On Fig 4B, the point at 0 mm is extrapolated and not measured. The chamber was placed in Zeiss Intrabeam water phantom in which the chamber is fixed and placed in a waterproof sleeve. The PDD is measured by moving the applicator away from the chamber.

In this study, gel PDD was compared with ionization chamber PDD in order to verify the accuracy of the gel as a dosimeter.

## Results

### Soft tissue equivalence at low energy

Calculated mass attenuation coefficient (μ/ρ), mass energy-absorption coefficient (μ_en_/ρ), mass stopping power (S/ρ) and mass scattering power (T/ρ) of the gel are shown in Table 1. Values around 1 indicated that radiological properties of the substitutes were similar to those of the biological tissues.

**Table 1:** Ratios between the electron and photon characteristic quantities (mass attenuation coefficient μ/ρ, mass energy-absorption coefficient μ_en_/ρ, mass stopping power S/ρ and mass scattering power T/ρ) of the ferrous gel versus the soft tissue reference for energies between 10keV and 50keV.

Energy (keV)	μ/ρ	μ_en_/ρ	(S/ρ)	(T/ρ)
10	**0.97**	**0.97**	1.00	1.01
20	0.97	0.96	1.00	1.01
30	0.98	0.95	1.00	1.01
40	0.99	0.96	1.00	1.01
50	0.99	0.96	1.00	1.01

The gel used in this study had the same coefficients for electronic mass stopping power (S/ρ) and mass scattering power (T/ρ) as soft tissue for energies between 10 and 50keV. Mass energy absorption coefficient (μ_en_/ρ) and mass attenuation coefficient (μ/ρ) were also very close to soft tissue with 5% deviation maximum.

This gel can therefore be used as soft tissue equivalent for low energy dosimetry.

### INTRABEAM measurements

**Fig 3:**
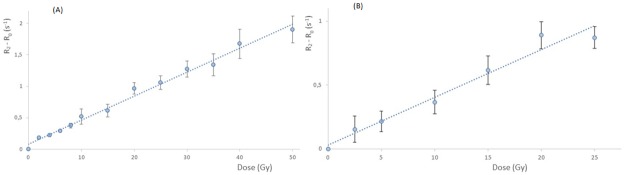
Gel dose response. (A) Measurement at low energy for 12 irradiation dose levels (2, 4, 6, 8, 10, 15, 20, 25, 30, 35, 40 and 50 Gy). The equation of the linear regression (dotted line) is $\text{R}2-{\text{R}}_{2}{}_{{}_{0}}=0.0381*\text{Dose}+0.0836({\text{r}}^{2}=0.9893)$. (B) Measurement at high energy for 6 irradiation dose levels (2.5, 5, 10, 15, 20 and 25 Gy). The equation of the linear regression (dotted line) is $\text{R}2-{\text{R}}_{2}{}_{{}_{0}}=0.0372*\text{Dose}+0.0326({\text{r}}^{2}=0.9662)$.

The calibration curve determined at low energy for the 4 cm applicator was a linear function (r^2^ = 0.9893) with a sensitivity of 0.0381 s^-1^.Gy^-1^ (Fig 3A). The SD average of (${\text{R}}_{2}-R_{2}{}_{{}_{0}}$) parameter was 0.091 s^-1^.

**Fig 4:**
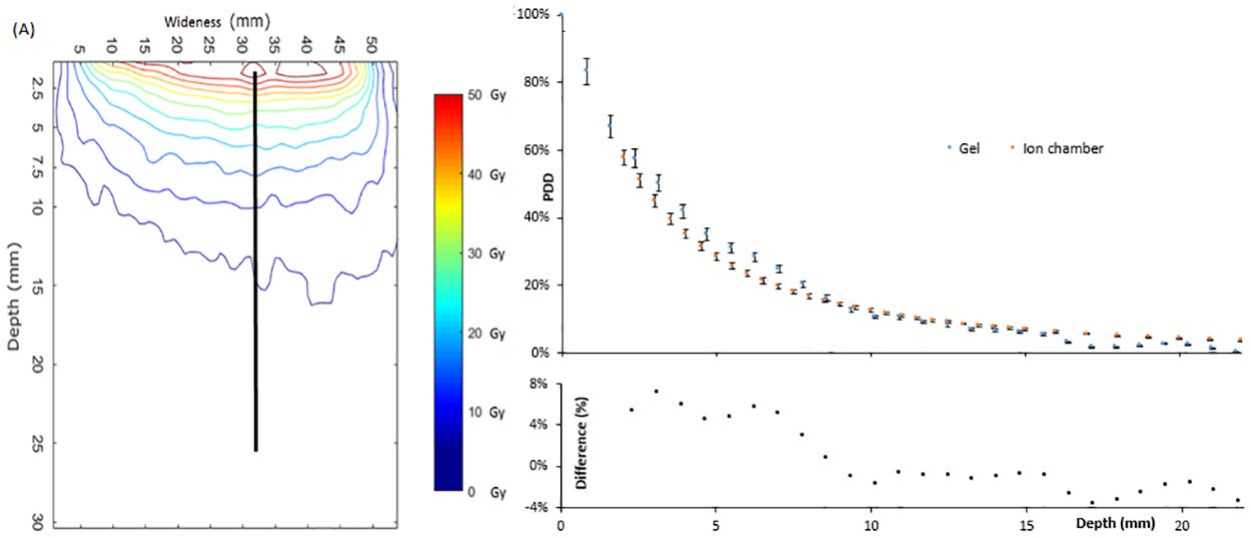
Dose distribution in gel. (A) Isodose map obtained for the irradiation dose level of 50 Gy. The black line defines the position of the Percent Depth Dose (PDD). (B) Normalized PDD measured from gel (blue) and from ion chamber (orange) with respective error bars. Indicative point of normalization at 0 mm for the ion chamber is obtained by extrapolation with an exponential fit.

The calibration curve was applied to the 50 Gy irradiation image to extract the dose distribution (Fig 4A). The maximum dose was detected at the gel surface then the dose decreased with depth and there was no residual dose after 20 mm. The pixel with the maximum dose was not found on the beam central axis but shifted by a few millimeters. One explanation of this asymmetric distribution could be related to the gel elasticity and the INTRABEAM arm weight: indeed, because of the arm weight, the applicator tended to distort gel surface, generating asymmetry in dose distribution. The PDD was determined from the detected surface maximum to a depth of 22 mm. For each pixel, the dose was reported from the isodose map and compared with ion chamber (Fig 4B). Error bars for gel measurements were derived from the standard deviation determined for ($R_{2}-R_{2}{}_{{}_{0}}$) and were equal to 4.8% of the dose value. For ion chamber measurements, a 4% error bar was applied to account for the uncertainty in detector calibration factor given on the certificate. In order to calculate the difference between the plots, it was necessary to interpolate the data as the data z spacing was different. Ion chamber measurements were fit to the following equation (generic formula used by Zeiss when commissioning source/applicator couples):

$$\text{PDD}=\text{exp}(1.839-0.841{\text{d}}^{0.5}+4479{\text{d}}^{-2.5})$$ (1)

Where d = z + 21.5 and represents the distance to the source in mm and z the distance to the applicator surface (in mm).

The absolute dose difference varies between -3.6 and + 7.2%. Differences higher than 5% can be explained by the gradient. It is common in radiotherapy to also look at the distance to agreement in high dose regions. Only one point (z = 6.24 mm) of the gel curve is not within 1 mm of ion chamber plot. The plot differences are therefore deemed acceptable.

### High energy measurements

At high energy, the gel presented a sensitivity of 0.0372 s^-1^.Gy^-1^ and a correlation coefficient of r^2^ = 0.9662 (Fig 3B). As for low energy, a linear function was found for the calibration curve. The uncertainties for the measured relaxation rates at the low dose levels appeared to be higher than those for the INTRABEAM low energies.

The sensitivities at low and high energy were very similar, 0.0381 and 0.0372 s^-1^.Gy^-1^ respectively, showing that the gel sensitivity did not depend much on photon beam energy.

## Discussion

The Carl Zeiss Surgical INTRABEAM system (Oberkochen, Germany) is a miniature accelerator producing low energy photons (50 keV maximum). The only published dosimetric characterization of the INTRABEAM system for flat and surface applicators was based on radiochromic films or ionization chambers [26,27].

The objective of this work was to determine the sensitivity of a paramagnetic ferrous gel with MR imaging in low energy x-ray beam using Carl Zeiss Surgical INTRABEAM system. Results showed that the relation between the dose and the relaxation rate was a linear function with a gel sensitivity of 0.0381 s^-1^.Gy^-1^ (r^2^ = 0.9893). The ferrous gel used in this study has a lower dose sensitivity than the MAG (Methacrylic Acid Gelatin) (0.04 vs. ~1. s^-1^. Gy^-1^) with a more important range of linearity (0–50 Gy vs. 0–20 Gy). This is the first time that such results are presented for low energy x-ray beam. Furthermore, the gel sensitivity at low and high energy was very close, showing a weak energy dependence of the gel sensitivity. It was important to perform high energy irradiation with this gel to compare results with previous studies using the same gel [20,21]. The weak energy dependence of the gel for these two very different energies suggests that a calibration at higher energy could be used for low energy. The advantage of using a higher energy for gel sensitivity calibration lies in the lower dose gradient at high energy, resulting in a more accurate dose determination.

The review of MacDougall *et al* put forward that the median accuracy for polymer gels is 10%, derivatives of Fricke gels have an accuracy of 5% and a precision of 1.5% [44]. The same overview showed that the accuracy worsens at lower absorbed doses. In our study, reproducibility measurements showed a variation of 9.7% (precision) which was higher than expected from MacDougall *et al* review [44]. This variation was also slightly higher than the value reported by Stien *et al* with the same gel but at high energy [21]. This difference could be related to the contact of the gel with water during gel handling (irradiation and MR imaging). The gel might have swelled resulting in an inhomogeneity on the surface. This might explain the higher variation in the precision results. Also, in our study, the fact that dose calibration curves were based on different images of different MR acquisitions might have added uncertainties in dose determination as well.

**Fig 5:**
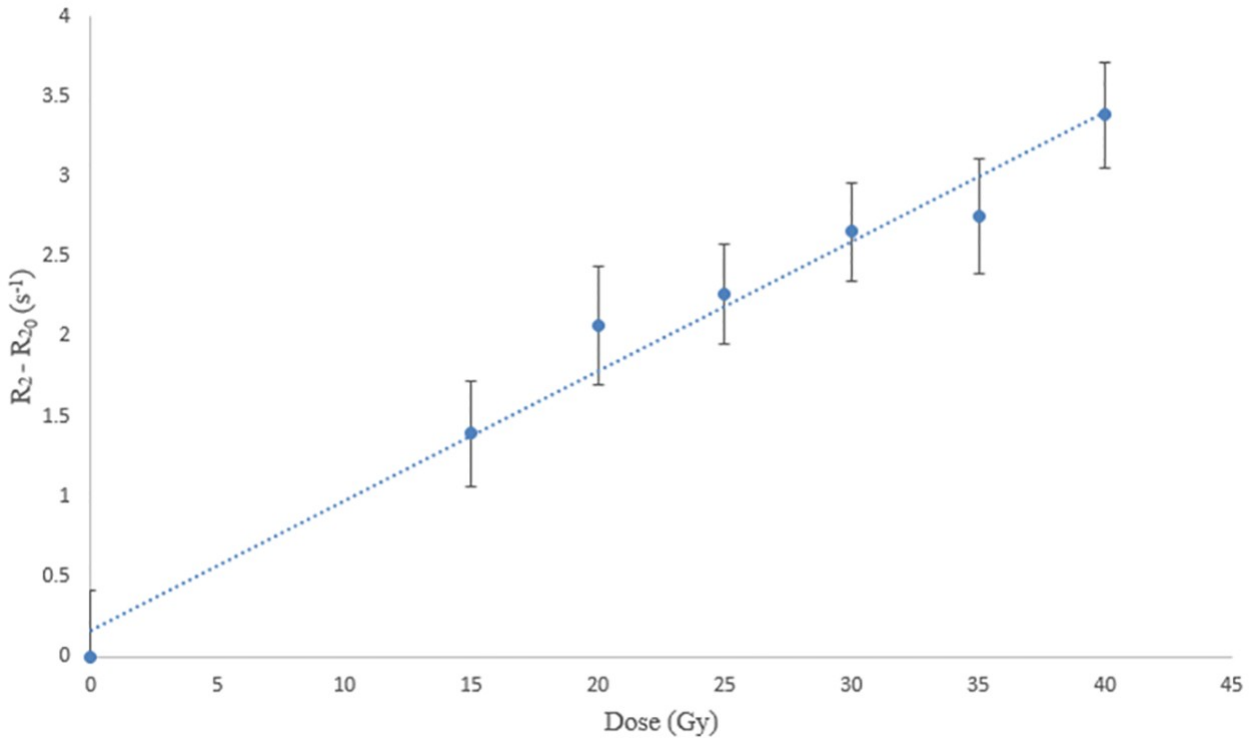
Gel dose response measured with vegetable oil at low energy for 6 irradiation dose levels (15, 20, 25, 30, 35 and 40 Gy). The equation of the linear regression (dotted line) is ${\text{R}}_{2}-{\text{R}}_{2}{}_{{}_{0}}=0.0811*\text{Dose}+0.1627({\text{r}}^{2}=0.9757)$.

In order to avoid gel swelling, as the gel has hydrophilic properties, water was replaced by vegetable oil (Fig 5).

The transverse relaxation rate R_2_ was close to 9 ms^-1^ with the gels immersed in vegetable oil. This value corresponds to the transverse relaxation rate of the vegetable oil [45]. The transverse relaxation rate R_2_ was close to 2 ms^-1^ with the gels immersed in water.

In an aqueous solution of ferric ions, water exists in two different environments. Some water molecules are in the coordination shells of ions (bound water molecules or hydration water molecules). The rest of the water molecules are the bulk (free water). The apparent spin relaxation rate is the weighted average of the inherent relaxation rates of the two groups [9]:

$$R_{2}{}_{{}_{app}}=p.R_{in}+\left(p-1\right)R_{w}$$ (2)

where p is the fraction of proton which are on the coordination of water molecules, *R*_*in*_ is the inherent relaxation rate of the protons on these coordination water molecules and *R*_*w*_ is the relaxation rate of the bulk water protons.

If the concentration of the magnetic species $C_{{Fe}^{3+}}$ is low enough to ensure no overlap between the coordination shells of neighboring ions, the coordination water fraction p is:

p=n.CFe3+Cw (3)

where n is the number of water molecules in actual contact with ion and *C*_*w*_ is the considerate water concentration.

The Solomon-Bloembergen equation was found [46,47] by rewriting Eq (2) as:

$$R_{2}{}_{{}_{app}}=R_{w}+r_{2}.C_{Fe^{3+}}$$ (4)

where r2=(nCw)(Rin-Rw)

When the gel is in contact with water, it swells as the water diffuses at the gel surface and a decrease of the gel sensitivity is observed due to the increase of the water concentration. The value of $R_{2}{}_{{}_{0}}$ is not affected ($R_{2}{}_{{}_{0}}=R_{w}$). If we consider that all the samples swell in the same way, differences will increase proportionally to the dose.

Water was replaced by vegetable oil to avoid gel swelling by water diffusion at gel surface but a different process occurred, also resulting in gel swelling. When the gel is in contact with vegetable oil, the oil is absorbed by the hydrophobic components of the gelatin. The increase of oil concentration at the surface causes a decrease of water concentration and an increase of gel sensitivity is observed. This time, the value of $R_{2}{}_{{}_{0}}$ is affected and it increases according to the vegetable oil proportion *p*_*h*_ which has penetrated into the gel ($R_{2}{}_{{}_{0(cal)}}=\left(1-p_{h}\right).R_{w}+p_{h}.R_{2}{}_{{}_{oil}}$). The reference $R_{2}{}_{{}_{0(mes)}}$ is taken at the bottom of the plastic containers and this zone is not affected by vegetable oil ($R_{2}{}_{{}_{0(mes)}}=R_{w}$). As $\Delta R_{2}{}_{{}_{0}}\gg \Delta r_{2}$, the total uncertainty corresponds to the variations of the $R_{2}{}_{{}_{0}}$ value, so there is an identical increase of variations for all doses. In addition, a deterioration of the gel 24 hours after immersion in vegetable oil was observed, making this approach limited in time.

In order to decrease the swelling effect, other solvents were tested to replace water (ethanol and butan-1-ol) but they presented drawbacks for the implementation (gel interaction for ethanol and unpleasant smell for butan-1-ol). It would be interesting to carry out measurements with other immersion liquids that would not interact with the gel and that would be convenient to use.

In order to avoid the repetition of several acquisitions due to multiple image sets, smaller containers (5 cm diameter and 0.5 cm depth capsules filled with gel) were used, allowing having all doses on the same image but they did not improve significantly the calibration curve accuracy. These results showed that the dose could be measured in different images with no impact on measurements reproducibility.

An isodose map was calculated from low energy measurements. The maximum was detected at the surface and there was almost no dose after 2 cm depth as it was shown in the study of Goubert *et al* with the films [27]. Dose distribution was found to be asymmetric. Goubert *et al* have already shown that the irradiation can be sometimes not perfectly isotropic (up to 8% difference) [27]. Other explanation could be found in the irradiation protocol during which the applicator tended to push the gel surface under the weight of the arm. To overcome this technical problem, a holding system could be used in order to center correctly the applicator and to keep it in contact of the surface of the gel. The isodose map was a relevant tool for the 2D dose distribution visualization. A PDD was also determined from gel measurements and the gel PDD is within 5%-1mm of the ion chamber PDD. At low energy, beam energy varied with depth. As the sensitivity curve was similar for the INTRABEAM low energy photon beam and 18 MV photon beam, it was reasonable to apply the same calibration curve for the entire image, and therefore ignoring energy variation with depth.

## Conclusion

This is the first time that ferrous gel sensitivity on MR imaging was investigated for INTRABEAM low energy photon beams. It was shown that the gel sensitivity is similar at low and high energy: the dose response can be represented by a linear function, with very close sensitivities. With this gel, it was possible to represent a PDD and the 2D dose distribution at low energy. Indeed, unlike ion chamber or film, the gel allows dose measurements in the first two millimeters.

Future work will consist in investigating other different immersion liquids, to formulate a new gel with a higher sensibility and measurements of the dose distribution produced by the other INTRABEAM applicators: surface applicator with different diameters, flat applicators, needle and spherical applicators.

## Supporting information

S1 Fig (DOCX)

S2 Fig (DOCX)

S3 Fig (DOCX)
