# Supplementary material for: Determination of paramagnetic ferrous gel sensitivity in low energy x-ray beam produced by a miniature accelerator
Source: PLoS One. 2020 May 4;15(5):e0232315. doi: 10.1371/journal.pone.0232315 (PMC7197784; doi:10.1371/journal.pone.0232315)
Supplement: S1 Fig — (DOCX) [file pone.0232315.s001.docx]

| Dose (Gy) | R_2_-R_0_ (s^-1^) |
| --- | --- |
| 0 | 0 |
| 2 | 0.00015447 |
| 4 | 7.33E-05 |
| 6 | 0.00020413 |
| 8 | 8E-05 |
| 10 | 0.0001724 |
| 15 | 0.000454 |
| 20 | 0.0003454 |
| 25 | 0.0007508 |
| 30 | 0.0008726 |
| 35 | 0.0010183 |
| 40 | 0.0010084 |
| 50 | 0.00133947 |

**Figure 3 (A)**

| Dose (Gy) | R_2_-R_0_ (s^-1^) |
| --- | --- |
| 0 | 0 |
| 2.5 | 0.15457322 |
| 5 | 0.21519946 |
| 10 | 0.36675997 |
| 15 | 0.61524842 |
| 20 | 0.89068206 |
| 25 | 0.87100158 |

**Figure 3 (B)**
