# Supplementary material for: Determination of paramagnetic ferrous gel sensitivity in low energy x-ray beam produced by a miniature accelerator
Source: PLoS One. 2020 May 4;15(5):e0232315. doi: 10.1371/journal.pone.0232315 (PMC7197784; doi:10.1371/journal.pone.0232315)
Supplement: S2 Fig — (DOCX) [file pone.0232315.s002.docx]

| Depth gel (mm) | PDD gel | Depth ion chamber (mm) | PDD ion chamber |
| --- | --- | --- | --- |
| 0 | 1 | 0 | 1.0000 |
| 0.78 | 0.83193549 | 2 | 0.5778 |
| 1.56 | 0.67000456 | 2.5 | 0.5114 |
| 2.34 | 0.57471407 | 3 | 0.4499 |
| 3.12 | 0.50180871 | 3.5 | 0.3962 |
| 3.9 | 0.41989099 | 4 | 0.3526 |
| 4.68 | 0.35083369 | 4.5 | 0.3160 |
| 5.46 | 0.30984671 | 5 | 0.2849 |
| 6.24 | 0.28330144 | 5.5 | 0.2574 |
| 7.02 | 0.24887175 | 6 | 0.2346 |
| 7.8 | 0.20370752 | 6.5 | 0.2139 |
| 8.58 | 0.16210195 | 7 | 0.1963 |
| 9.36 | 0.12787839 | 7.5 | 0.1807 |
| 10.14 | 0.10584363 | 8 | 0.1670 |
| 10.92 | 0.10418134 | 8.5 | 0.1546 |
| 11.7 | 0.09187606 | 9 | 0.1436 |
| 12.48 | 0.08205077 | 9.5 | 0.1337 |
| 13.26 | 0.07045893 | 10 | 0.1246 |
| 14.04 | 0.06588494 | 10.5 | 0.1166 |
| 14.82 | 0.06258498 | 11 | 0.1092 |
| 15.6 | 0.054934 | 11.5 | 0.1025 |
| 16.38 | 0.03254128 | 12 | 0.0962 |
| 17.16 | 0.01859535 | 12.5 | 0.0905 |
| 17.94 | 0.01738417 | 13 | 0.0854 |
| 18.72 | 0.02196914 | 13.5 | 0.0807 |
| 19.5 | 0.02584868 | 14 | 0.0761 |
| 20.28 | 0.02509476 | 14.5 | 0.0721 |
| 21.06 | 0.01462547 | 15 | 0.0684 |
| 21.84 | 0.00229002 | 16 | 0.0614 |
|  |  | 17 | 0.0556 |
|  |  | 18 | 0.0503 |
|  |  | 19 | 0.0458 |
|  |  | 20 | 0.0417 |
|  |  | 21 | 0.0381 |
|  |  | 22 | 0.0348 |

**Figure 4**
