# Supplementary material for: Determination of paramagnetic ferrous gel sensitivity in low energy x-ray beam produced by a miniature accelerator
Source: PLoS One. 2020 May 4;15(5):e0232315. doi: 10.1371/journal.pone.0232315 (PMC7197784; doi:10.1371/journal.pone.0232315)
Supplement: S3 Fig — (DOCX) [file pone.0232315.s003.docx]

| Dose (Gy) | R_2_-R_0_ (s^-1^) |
| --- | --- |
| 0 | 0 |
| 15 | 1.39331562 |
| 20 | 2.06682588 |
| 25 | 2.26386589 |
| 30 | 2.65428589 |
| 35 | 2.75188404 |
| 40 | 3.38309721 |

**Figure 5**
